# Preemptive pharmacogenetic testing to guide chemotherapy dosing in patients with gastrointestinal malignancies: a qualitative study of barriers to implementation

**DOI:** 10.1186/s12885-022-09171-6

**Published:** 2022-01-08

**Authors:** Kelsey S. Lau-Min, Lisa A. Varughese, Maria N. Nelson, Christine Cambareri, Nandi J. Reddy, Randall A. Oyer, Ursina R. Teitelbaum, Sony Tuteja

**Affiliations:** 1 Division of Hematology/Oncology, Department of Medicine, Perelman School of Medicine, University of Pennsylvania, Philadelphia, PA USA; 2 Division of Translational Medicine and Human Genetics, Department of Medicine, Perelman School of Medicine, Smilow Center for Translational Research, University of Pennsylvania, 3400 Civic Center Boulevard, Bldg. 421 11th Floor, Room 143, Philadelphia, PA 19104-5158 USA; 3 Gartner Consulting, Stamford, CT USA; 4 Department of Pharmacy, Hospital of the University of Pennsylvania, Philadelphia, PA USA; 5 Ann B. Barshinger Cancer Institute, Penn Medicine at Lancaster General Health, Lancaster, PA USA

**Keywords:** Pharmacogenetic testing, Chemotherapy dosing, Gastrointestinal cancers, Barriers to implementation

## Abstract

**Background:**

Pharmacogenetic (PGx) testing for germline variants in the *DPYD* and *UGT1A1* genes can be used to guide fluoropyrimidine and irinotecan dosing, respectively. Despite the known association between PGx variants and chemotherapy toxicity, preemptive testing prior to chemotherapy initiation is rarely performed in routine practice.

**Methods:**

We conducted a qualitative study of oncology clinicians to identify barriers to using preemptive PGx testing to guide chemotherapy dosing in patients with gastrointestinal malignancies. Each participant completed a semi-structured interview informed by the Consolidated Framework for Implementation Research (CFIR). Interviews were analyzed using an inductive content analysis approach.

**Results:**

Participants included sixteen medical oncologists and nine oncology pharmacists from one academic medical center and two community hospitals in Pennsylvania. Barriers to the use of preemptive PGx testing to guide chemotherapy dosing mapped to four CFIR domains: intervention characteristics, outer setting, inner setting, and characteristics of individuals. The most prominent themes included 1) a limited evidence base, 2) a cumbersome and lengthy testing process, and 3) a lack of insurance coverage for preemptive PGx testing. Additional barriers included clinician lack of knowledge, difficulty remembering to order PGx testing for eligible patients, challenges with PGx test interpretation, a questionable impact of preemptive PGx testing on clinical care, and a lack of alternative therapeutic options for some patients found to have actionable PGx variants.

**Conclusions:**

Successful adoption of preemptive PGx-guided chemotherapy dosing in patients with gastrointestinal malignancies will require a multifaceted effort to demonstrate clinical effectiveness while addressing the contextual factors identified in this study.

**Supplementary Information:**

The online version contains supplementary material available at 10.1186/s12885-022-09171-6.

## Background

Fluoropyrimidines such as 5-fluorouracil (5-FU) and capecitabine form the backbone of chemotherapeutic treatment for gastrointestinal malignancies, often in conjunction with irinotecan and/or oxaliplatin. Some patients are at increased risk for severe toxicity from fluoropyrimidines and irinotecan due to pharmacogenetic (PGx) variants in the *DPYD* (encoding dihydropyrimidine dehydrogenase, DPD) and *UGT1A1* (encoding uridine diphosphate-glucuronosyltransferase, UGT) genes, respectively [1–3]. Prospective studies have demonstrated that preemptive genotyping for *DPYD* variants can reduce chemotherapy-related toxicity and improve patient safety, and many guidelines now recommend that patients with *DPYD* or *UGT1A1* variants should undergo genotype-guided dose reductions when treated with fluoropyrimidines or irinotecan [2, 4–7].

Despite the known association between PGx variants and chemotherapy toxicity, preemptive testing prior to chemotherapy initiation is rarely performed in practice. Multiple barriers to implementation have been proposed including a lack of prospective trials supporting genotype-guided treatment algorithms, lengthy PGx test turnaround times, variable result reporting, clinician inexperience with PGx test interpretation, and cost considerations [8]. However, few studies have focused specifically on the use of PGx testing in oncology, a field in which the balance between chemotherapeutic efficacy and drug toxicity is paramount. As such, we conducted a qualitative study of oncology clinicians to identify barriers to using PGx testing to guide chemotherapy dosing in patients with gastrointestinal malignancies.

## Methods

### Study setting, sampling, and recruitment

Semi-structured interviews were conducted between October 2019 and March 2020 among clinicians from three Penn Medicine hospitals, including an urban academic medical center, an urban community hospital, and a rural hospital. At the time of this study, *DPYD* and *UGT1A1* genotyping was available through commercial laboratories, but preemptive testing to guide chemotherapy dosing was not standard practice. Census sampling was used to identify all medical oncologists and oncology pharmacists involved in the treatment of gastrointestinal malignancies. Respondents were recruited via e-mail until reaching thematic saturation (the point at which additional interviews do not produce further insights) [9, 10]. Thirty clinicians were invited to participate, of whom 25 (83%) consented and completed the interview. The remaining five clinicians did not respond to the recruitment e-mail.

### Data collection

A semi-structured interview guide was developed and included questions on prior experiences with PGx testing, barriers to using preemptive PGx testing to guide chemotherapy dosing, and feedback on a sample PGx result report adapted from established guidelines [2, 7]. The interview guide was informed by the Consolidated Framework for Implementation Research (CFIR) [11], an established approach to evaluating implementation context that incorporates factors such as intervention characteristics, the inner setting (characteristics of the implementing organization), the outer setting (external influences on implementation), characteristics of individuals, and the process of implementation. Prior to data collection, we pilot tested our interview guide with one oncology clinician whose responses were not included in the final analysis. After providing verbal informed consent, all respondents completed a brief survey containing demographic questions and assessing their baseline comfort level with interpreting and using PGx test results to guide chemotherapy dosing. They were then interviewed by one of the study authors (KSL or LAV). KSL is a hematology/oncology fellow who had previously interacted with a subset of the respondents in educational and clinical settings. LAV is a pharmacogenomics postdoctoral fellow who had no relationship with the respondents prior to the study. Both KSL and LAV were trained in the use of qualitative research techniques by the Mixed Methods Research Laboratory at the University of Pennsylvania. Interviews were conducted in-person or via telephone and were audio recorded with permission. Field notes were recorded during and after each interview. Following interview completion, all data were transcribed verbatim (Datagain, Secaucus, NJ) and de-identified.

### Data analysis

**Fig. 1:**
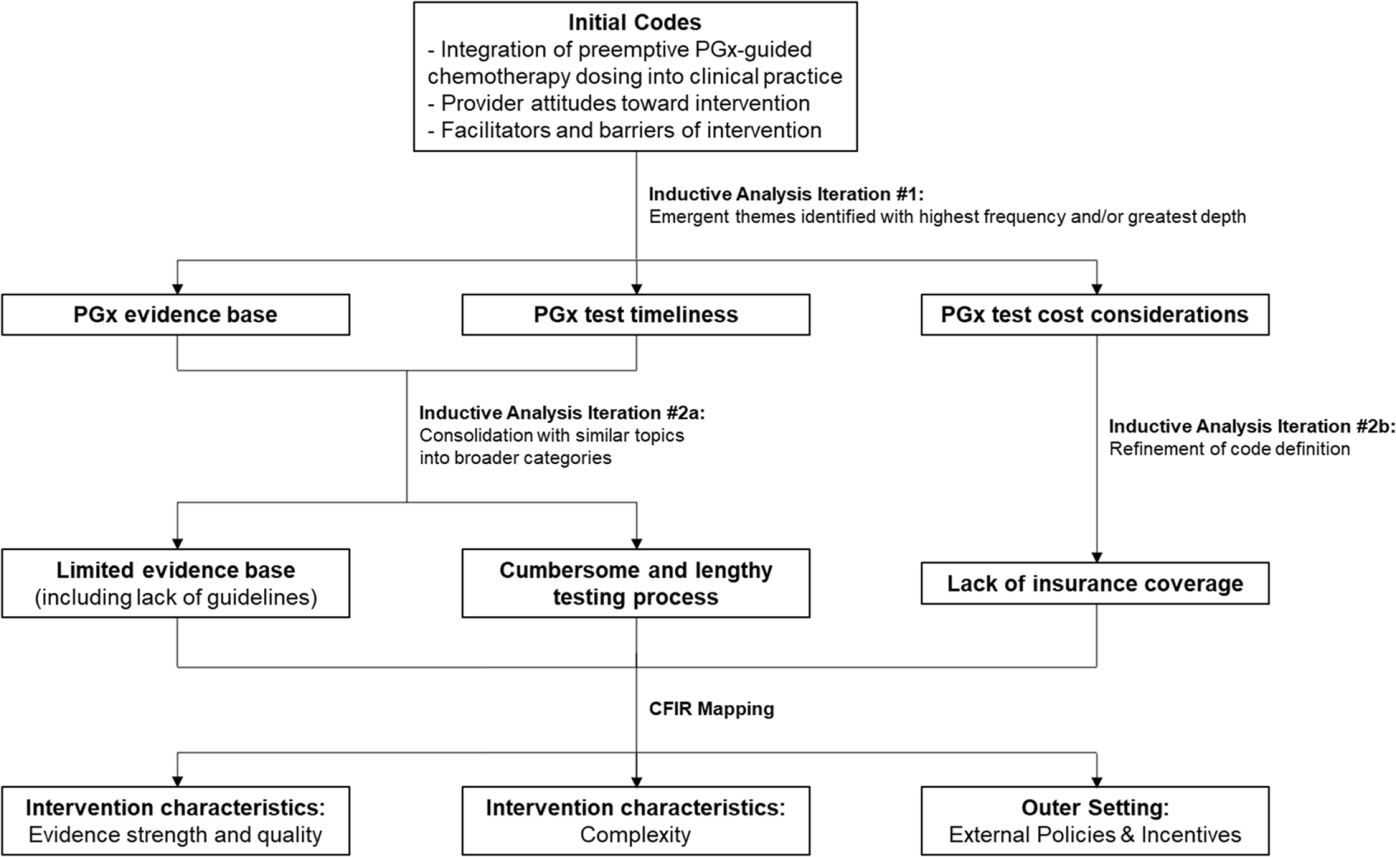
Schematic of inductive content analysis approach used to iteratively code interview transcript. Figure depicts three exemplar themes: 1) a limited evidence base, 2) a cumbersome and lengthy testing process, and 3) a lack of insurance coverage for preemptive PGx testing. PGx = pharmagonetic. CFIR = Consolidated Framework for Implementation Research

Interview transcripts were uploaded to NVivo v.12 to support coding and analysis. Two coders (KSL, LAV) used an inductive content analysis approach (Fig. 1) to iteratively analyze the interviews and develop a coding scheme that incorporated elements of the CFIR, the Genomic Medicine Integrative Research (GMIR) Framework, and themes that emerged as the data were collected (see Additional file 1) [11–14]. During this process, individual coders coded early transcripts independently to identify patterns in the data, including themes that emerged with notable frequency or depth. Team meetings were then used to explore the data line-by-line to reach consensus on emerging topics, address identified discrepancies, collapse similar topics into broader categories, and refine the codebook to be used in the analysis. The researchers then applied the codebook to the data and established strong inter-rater reliability, Κ = 0.92 with 11 (44%) interviews. The remaining 14 interviews were divided between the reviewers and coded independently. Finally, themes were mapped back to CFIR domains and constructs to facilitate study interpretation and applicability to eventual implementation efforts.

This study was deemed exempt by the University of Pennsylvania Institutional Review Board. The consolidated criteria for reporting qualitative research (COREQ) framework was used to guide research reporting [15].

## Results

### Respondent characteristics

Twenty-five clinicians participated in this study – 16 (64%) were medical oncologists and 9 (36%) were oncology pharmacists (Table 1). The mean age was 41, and most respondents were female (56%) and non-Hispanic white (72%). Most respondents reported feeling comfortable or very comfortable with interpreting and using PGx test results to guide chemotherapy dosing. Interviews ranged from 23 to 41 min (mean 30.3 min).

**Table 1:** Respondent characteristics

Characteristic	Frequency (%)
Number of respondents	25
Role	
Medical oncologist	16 (64%)
Oncology pharmacist	9 (36%)
Mean age, years (range)	41.2 (28–67)
Sex	
Male	11 (44%)
Female	14 (56%)
Race	
Non-Hispanic White	18 (72%)
Asian	7 (28%)
Mean time in practice, years (range) ^*a*^	10.2 (0.5–35)
Comfort with interpreting PGx test results	
Comfortable or very comfortable	15 (60%)
Neutral	3 (12%)
Uncomfortable or very uncomfortable	7 (28%)
Comfort with using PGx test results to guide chemotherapy dosing	
Comfortable or very comfortable	14 (56%)
Neutral	4 (16%)
Uncomfortable or very uncomfortable	7 (28%)
Mean interview duration, minutes (range)	30.3 (23–41)
Interview modality	
In-person	14 (56%)
Telephone	11 (44%)

^*a*^ Not including residency or fellowship training

### Barriers to Preemptive PGx-Guided Chemotherapy Dosing.

Barriers to using preemptive PGx testing to guide chemotherapy dosing clustered into four of the five CFIR domains: intervention characteristics, outer setting, inner setting, and characteristics of individuals (Table 2). All but one of the barriers were reported by medical oncologists and oncology pharmacists alike.

**Table 2:** Barriers to the implementation of preemptive pharmacogenetic-guided chemotherapy dosing

CFIR Domain	CFIR Construct	Theme	Representative Quotation
Intervention characteristics	Evidence strength and quality	Limited evidence base	For therapeutic dose recommendations, I want it to be from NCCN, ASCO, or the Europeans … Anything less than that is weak. (O9) I have no problems with palliative intent … but when we talk about curative, unless we had prospective data, I’d be a little more cautious. (O15)
	Relative Advantage	Questionable impact on clinical care	How often is this really going to change our therapy? ...If we don’t change it, is it a matter of life or death, or is it slight toxicity that we can manage otherwise? (P3)
	Complexity	Cumbersome and lengthy testing process	If you don’t make it … as easy as a CBC … you may get a start, but somewhere down the road, it will fall off the track. (O12) I don’t – I can’t – do preemptive [PGx testing] because usually when they’re coming to see me, they need to start therapy sometime in the next couple of weeks, and so I’m not going to have a result back to actually do a meaningful assessment. (O1)
Outer Setting	Patient Needs & Resources	Lack of alternative therapeutic options^a^	If you don’t have 5-FU for a colon cancer patient, you really are very limited in terms of what you have in your treatment armamentarium, especially if they have a curable disease … (O3)
	External Policies & Incentives	Lack of insurance coverage	I would certainly want to know what, if any, cost or coinsurance was passed down to the patient … I’d be a little bit worried if it turned out that only people with certain types of insurance would be eligible for the testing. (O8)
Inner Setting	Access to Knowledge & Information	Challenging PGx test interpretation	Once I had the information, *UGT1A1*, for instance, I wouldn’t know what to comfortably do with it. (O7) If you’re in a non-academic center and you don’t have other people with expertise in a multidisciplinary fashion, then you’re sort of stuck with a test that you’re not sure what to do with. (O14)
Characteristics of Individuals	Knowledge & Beliefs About the Intervention	Clinician lack of knowledge	I honestly perceive just a baseline deficiency in my own education around *DPYD*. (P7)
	Self-efficacy	Remembering to order PGx testing for eligible patients	The prescriber remembering to do it … is always the worst. (P2)

^a^ Reported by medical oncologists but not oncology pharmacists

*Abbreviations*: *CFIR* Consolidated Framework for Implementation Research, *O* Medical oncologist, *NCCN* National Comprehensive Cancer Network, *ASCO* American Society of Clinical Oncology, *P* Oncology pharmacist, *PGx* Pharmacogenetic, *5-FU* 5-fluorouracil

### Intervention characteristics

#### Limited evidence base

One of the most frequently cited concerns about preemptive PGx-guided chemotherapy dosing was a perceived lack of evidence for this practice. Respondents noted that the best practice guidelines most commonly used by clinical oncologists, such as those issued by the National Comprehensive Cancer Network (NCCN), American Society of Clinical Oncology (ASCO), and European Society for Medical Oncology (ESMO), do not currently recommend the use of preemptive PGx testing to guide chemotherapy dosing; as such, they do not feel compelled to use this strategy in practice. They expressed a great deal of trust in these national guideline bodies, presuming that a lack of support for preemptive PGx testing was supported by published literature and expert consensus. They did not, however, place the same degree of trust in other, less familiar entities such as the Clinical Pharmacogenetics Implementation Consortium (CPIC).

Balancing drug toxicity against efficacy was another challenge. Clinicians recognized the potential for preemptive PGx-guided chemotherapy dosing to decrease chemotherapy-related toxicity. As a result, they reported feeling more comfortable using this strategy for patients with advanced or metastatic disease given that these patient’s goals of care are focused more on quality of life and symptom management rather than curing their cancer. However, the impact of preemptive PGx-guided chemotherapy dosing on clinical oncologic outcomes has not been prospectively studied to date, so clinicians expressed concerns that this strategy could result in decreased drug efficacy for patients treated with curative intent. They wanted to be reassured that using a preemptive PGx-guided approach would result in equivalent outcomes to the current standard of care before implementing it into their practice for patients with earlier-stage disease.

#### Questionable impact on clinical care

Participants questioned the impact that preemptive PGx-guided chemotherapy dosing would have on clinical care and whether it would indeed offer an advantage over their existing practice patterns. They described the test as unreliable, citing instances when patients with severe chemotherapy-related toxicity were found to have negative PGx test results and vice versa. They also expressed uncertainty about whether preemptively knowing a patient’s PGx status would change management, as clinicians reported already having established approaches to recognizing and mitigating chemotherapy-related toxicities with dose reductions, delays, and other symptom management strategies. Clinicians did not believe that knowledge of a patient’s PGx test result would necessarily change this overarching approach to care.

### Cumbersome and Lengthy Testing Process

Many clinicians described a PGx testing process that was perceived to be too burdensome and lengthy to be able to positively impact clinical care. At the time of this study, PGx testing at each study site was offered as a send-out test to external commercial laboratories. As a result, there was a great deal of confusion for multiple parties: clinicians did not know how to place PGx test orders in the electronic health record, phlebotomists were uncertain which tubes to use for patient specimen collection, and laboratory personnel were unsure of the workflows that were needed to send specimens to the appropriate laboratory. As a result, clinicians reported that some PGx test results simply never returned. Optimizing the PGx testing workflow was viewed as a key component to the implementation and long-term sustainability of preemptive PGx-guided chemotherapy dosing in clinical practice.

However, even if the PGx testing workflow were optimized, clinicians feared that results would not return quickly enough to impact clinical care. They stated that many patients with gastrointestinal malignancies are very ill at the time of diagnosis and need to start therapy as soon as possible. Most agreed that a PGx test turnaround time longer than 1 week would be infeasible if it were to be used to preemptively guide chemotherapy dosing.

### Outer setting

#### Lack of Alternative Therapeutic Options.

The one barrier that was reported by medical oncologists but not oncology pharmacists was the concern that knowledge of a patient’s PGx status may actually limit therapeutic options or complicate the management of some patients. Fluoropyrimidines are the backbone of the most effective chemotherapy regimens for patients with colorectal cancer, so the preemptive discovery of an actionable *DPYD* variant presents oncologists with a challenging decision between dose-reducing or omitting the fluoropyrimidine (and potentially sacrificing drug efficacy, as is described above) and proceeding with the knowledge that a patient may be at increased risk for toxicity. Some oncologists indicated that this kind of decision-making was so divergent from their existing treatment paradigm that they may simply prefer not to know of a patient’s PGx status before initiating chemotherapy.

#### Lack of Insurance Coverage.

One of the most prominent clinician concerns was that insurance companies would not fully cover PGx testing in the preemptive setting, in part because it is not currently incorporated into the national guideline recommendations that are used in insurance coverage determinations. As a result, they worried that preemptive PGx testing would lead to undue financial burden on patients, disparities in care, and differential clinical outcomes by insurance status.

### Inner setting

#### Challenging PGx test interpretation

Clinicians worried about their ability to interpret preemptive PGx test results. Some posited that standardized guidelines for genotype-guided chemotherapy dosing would be a useful strategy, though none of the respondents reported familiarity with existing CPIC guidelines that were specifically developed to accomplish this goal. In the event of more complex cases, respondents expressed interest in having specialist support for PGx test interpretation, a role that could be effectively filled by oncology pharmacists who were already well integrated into their clinical oncology practices. However, concerns were raised that the level of expertise among oncology pharmacists may vary depending on whether they practiced in academic versus non-academic settings, which in turn could lead to greater challenges with PGx test interpretation for clinicians in community oncology practices.

### Characteristics of individuals

#### Clinician lack of knowledge

Clinicians cited a general lack of knowledge as a barrier that could impact not only their initial decision to perform PGx testing but also their ability to counsel patients on the risks, benefits, and alternatives to preemptive PGx-guided chemotherapy dosing. Some stated that they had only received a cursory overview of PGx testing during their schooling or clinical training and would be interested in receiving additional education on this topic.

#### Remembering to order PGx testing for eligible patients

Even for clinicians who felt more well-versed, remembering to order preemptive PGx testing posed another challenge. Respondents shared that unless preemptive PGx testing were incorporated into their clinical workflows at the time of a patient’s initial diagnosis or consultation, the time and cognitive constraints of a busy clinic schedule would likely limit their ability to remember to order the test later in a patient’s clinical course. However, there was no consensus on whether universal PGx testing should be offered to all patients with gastrointestinal malignancies, including those who do not need fluoropyrimidine or irinotecan-based chemotherapy at the time of initial diagnosis.

## Discussion

In this study, we assessed clinician perspectives on using preemptive PGx testing to guide chemotherapy dosing in patients with gastrointestinal malignancies. Barriers to clinical implementation mapped to four CFIR domains including intervention characteristics, outer setting, inner setting, and characteristics of individuals. The three most prominent barriers included 1) a limited evidence base, 2) a cumbersome and lengthy testing process, and 3) a lack of insurance coverage for preemptive PGx testing.

Although multiple studies have identified similar barriers to implementing PGx testing in diverse clinical settings, few have focused specifically on the use of PGx testing in oncology [16–19]. One study by Martens and colleagues elicited perspectives on preemptive DPD testing among oncologists, pharmacists, laboratory specialists, and patients in the Netherlands; they identified many of the same barriers surrounding intervention characteristics and workflow considerations as we did [20]. Our study adds to the current body of literature by providing additional insight from oncology clinicians practicing within academic, urban community, and rural settings in one region of the United States.

One barrier to using preemptive PGx testing to guide chemotherapy dosing was a perceived lack of evidence. Multiple studies have demonstrated decreased chemotherapy-related toxicity with preemptive PGx testing, including two prospective trials that evaluated the impact of *DPYD* genotype-guided fluoropyrimidine dosing [4, 5]. However, less work has been done to assess the impact of this strategy on clinical oncologic outcomes. In one analysis, DPYD*2A heterozygotes treated with a 50% fluoropyrimidine dose reduction experienced non-inferior survival outcomes compared to matched DPYD*2A wild-type controls treated with standard dose chemotherapy [21]. Additional research is needed to confirm these findings in the prospective setting – multiple studies are ongoing and are anticipated to provide additional insight on this important question [22, 23].

There was also concern that the current PGx testing process is too cumbersome and lengthy to be effectively implemented into clinical care. The electronic health record (EHR) has emerged as an important tool for integrating genomic medicine into patient care, and many efforts focused on PGx testing are ongoing [24, 25]. EHR applications such as default PGx test orders, discrete reporting of variant results, and clinical decision support to guide chemotherapy dosing may be particularly effective in overcoming the workflow-related barriers cited in our study [26]. Additional research is needed to evaluate the effects of these EHR-based strategies on the uptake of preemptive PGx-guided chemotherapy dosing in routine practice.

Finally, study respondents cited lack of insurance coverage for preemptive PGx testing as a barrier to implementation, as many patients are reluctant to pay out-of-pocket for PGx testing [27]. Cost analyses of the two prospective trials on preemptive *DPYD*-guided fluoropyrimidine dosing in Europe showed that this strategy was at least cost-neutral after accounting for decreased healthcare utilization for chemotherapy-related toxicities [4, 28]. Subsequent modeling studies using United States-based cost data demonstrated preemptive *DPYD* and *UGT1A1* testing to be cost-effective in both the metastatic and adjuvant settings [29, 30]. The United States Centers for Medicare and Medicaid Services recently posted a Local Coverage Determination to cover PGx testing for medications with known drug-gene interactions, and commercial insurers are beginning to follow suit [31, 32].

This study has several limitations. First, because it was conducted within a single health system in Pennsylvania, our findings may not be generalizable to other practices. Second, it is possible that clinicians who agreed to participate in this study may have differed from nonrespondents in ways that may have influenced their reported perceptions on PGx testing. Finally, participants may have provided more favorable responses to please the interviewers, both of whom were trainees at the same institution. However, we believe the impact of social desirability bias was minimal since respondents shared a range of perspectives on PGx testing, both positive and negative.

## Conclusions

Preemptive PGx-guided chemotherapy dosing is a promising strategy to mitigate chemotherapy-related toxicity and improve patient safety. This study provides valuable insight on barriers that should be addressed as efforts are undertaken to implement this approach in clinical practice. Successful adoption of this strategy will require additional research demonstrating clinical effectiveness, seamless integration into existing clinical workflows, and broad dissemination of evolving guidelines and policies surrounding preemptive PGx testing.

## Supplementary Information

**Additional file 1.** Includes the codebook that was used to analyze study interview transcripts.

## Data Availability

The datasets generated and analyzed during the current study are not publicly available to ensure participant confidentiality, as was stated in the Institutional Review Board approval, but are available in de-identified manner from the corresponding author on reasonable request.

## Abbreviations

ASCO: American Society of Clinical Oncology
5-FU: 5-fluorouracil
CFIR: Consolidated Framework for Implementation Research
COREQ: Consolidated criteria for reporting qualitative research framework
EHR: Electronic health record
GMIR: Genomic Medicine Integrative Research framework
NCCN: National Comprehensive Cancer Network
PGx: Pharmacogenetics
