# Supplementary material for: Preemptive pharmacogenetic testing to guide chemotherapy dosing in patients with gastrointestinal malignancies: a qualitative study of barriers to implementation
Source: BMC Cancer. 2022 Jan 8;22:47. doi: 10.1186/s12885-022-09171-6 (PMC8742388; doi:10.1186/s12885-022-09171-6)
Supplement: Supplementary file 1 — Additional file 1. Includes the codebook that was used to analyze study interview transcripts. [file 12885_2022_9171_MOESM1_ESM.docx]

**Additional file 1.** Codebook used to analyze study interview transcripts.

| **Code** | **Definition** | **Notes** |
| --- | --- | --- |
| **Topic Codes ^a^** | | |
| Current chemotherapy dosing/verification practice | Use when participant discusses his/her approach to chemotherapy dosing or verification | Can include mention of new chemotherapy starts or dose modifications over time  Can also include mention of PGx testing if participant uses it in his/her current practice |
| Prior experience with PGx testing | Use when participant mentions previous experiences with PGx testing, either in the preemptive or reactive setting | Can include mention of prior education about PGx testing |
| Integration of intervention^b^ into clinical practice | Use when participant discusses how preemptive PGx-guided chemotherapy dosing can be integrated into his/her practice, either in routine clinical practice or as part of a prospective study | Can include mention of potential usefulness or indications for testing, ideal strategies for integration into patient care  *Note that preemptive PGx-guided chemotherapy dosing is not yet a part of standard clinical practice* |
| Sample PGx report feedback | Use when participant provides feedback on the format of the sample PGx test result and dose recommendation report |  |
| Prospective study considerations – design | Use when participant mentions suggestions for prospective study design | Can include mention of patient inclusion/exclusion criteria or randomization procedures |
| Prospective study considerations – outcomes | Use when participant mentions suggestions for prospective study outcomes | Can include mention of clinical, pharmacokinetic, or implementation outcomes |
| Additional comments | Code to question | Can include post-interview Q&A’s, though that content should also be coded to other topic and cross codes if appropriate |
| **Cross Codes ^a^** | | |
| Provider attitudes towards intervention | Use when participant mentions a positive, negative, or neutral preference, opinion, or sentiment on preemptive PGx-guided chemotherapy dosing | Can include provider attitudes towards preemptive PGx-guided chemotherapy dosing mentioned in any section of the interview |
| **Sub-code**: Positive sentiment | Use when participant mentions a preference, opinion, or sentiment that has a positive connotation | Can include expressions of support for the intervention, excitement, etc. |
| **Sub-code**: Negative sentiment | Use when participant mentions a preference, opinion, or sentiment that has a negative connotation | Can include expressions of hesitation toward the intervention, skepticism, etc. |
| **Facilitators and Barriers of Preemptive PGx-Guided Chemotherapy Dosing** | | |
| Facilitators of intervention | Use when participant discusses facilitators of incorporating preemptive PGx-guided chemotherapy dosing into clinical practice |  |
| Barriers to intervention | Use when participant mentions barriers or challenges to incorporating preemptive PGx-guided chemotherapy dosing into clinical practice |  |
| **PGx Test Characteristics** | | |
| PGx test timeliness | Use when participant mentions PGx test turnaround time | Separate from the timing of PGx testing in a patient’s clinical course (should be coded to “Integration of intervention into clinical practice” topic code above) |
| PGx test cost considerations | Use when participant discusses costs related to PGx testing | Can include mention of insurance reimbursement, health disparities |

| **Additional file 1**, continued. | | |
| --- | --- | --- |
| **Stakeholder Roles and Perspectives** | | |
| Medical oncologist/advanced practice provider role | Use when participant mentions the role of the medical oncologist (eg: MD, DO) or advanced practice provider (eg: physician assistant, nurse practitioner) in preemptive PGx-guided chemotherapy dosing | Should refer to a specific action on the part of the medical oncologist/APP  Can include mention of collaboration with other providers |
| Pharmacist/PGx specialist role | Use when participant mentions the role of the oncology pharmacist or PGx specialist in preemptive PGx-guided chemotherapy dosing | Should refer to a specific action on the part of the pharmacist/PGx specialist  Can include mention of collaboration with other providers |
| Patient perspectives | Use when participant discusses his/her perception of patient perspectives on preemptive PGx-guided chemotherapy dosing or prospective study design | Can include mention of the patient perspective on prospective study randomization |
| **Other Considerations** | | |
| EHR integration | Use when participant mentions strategies to integrate any part of the preemptive PGx-guided chemotherapy dosing process in the electronic health record, either in routine clinical practice or part of a prospective study | Can include mention of PGx test ordering, results reporting, results notification, clinical decision support, screening patients to identify those appropriate for testing  Separate from PGx report format (should be coded to “Sample PGx report feedback” topic code above) |
| PGx evidence base | Use when participant discusses the evidence base for preemptive PGx-guided chemotherapy dosing | Can include mention of prior studies, published literature, guideline recommendations  Should not simply include mention of references in the sample PGx report (should be coded to “Sample PGx report feedback” topic code above) |
| Baseline PGx knowledge deficit | Use when participant discusses his/her lack of baseline knowledge about PGx or discusses genomic testing that is clearly not related to PGx | Can include mention of germline genetic testing for inherited cancer syndromes |
| Other provider attitudes | Use when participant discusses a positive, negative, or neutral preference, opinion, or sentiment that is not related to the intervention | Separate from preferences, opinions, or sentiments related to the intervention (should be coded to “Provider attitudes towards intervention” cross-code above) |
| Miscellaneous | Use when a participant mentions something not captured by any of the other codes |  |
| ^a^ Content can be cross-coded between topic and cross-codes (eg: topic and cross-codes are not mutually exclusive) | | |
| ^b^ Any mention of “intervention” refers to preemptive pharmacogenetic (PGx)-guided chemotherapy dosing | | |
